# Comparative Evaluation of Platelet-Rich Fibrin and Mineral Trioxide Aggregate in the Direct Pulp Capping of Carious Exposures: A Randomized Clinical Trial

**DOI:** 10.7759/cureus.108203

**Published:** 2026-05-03

**Authors:** Geeta Asthana, Rajashree Tamuli, Sadhna Manglani, Saloni Mandhare, Ragini Kulkarni, Anooja Mathirat, Parneet Kaur, P Jyothirmayee, Surabhi Landge

**Affiliations:** 1 Department of Conservative Dentistry and Endodontics, Government Dental College and Hospital, Ahmedabad, Ahmedabad, IND

**Keywords:** autologous biomaterial, cone-beam computed tomography, direct pulp capping, mineral trioxide aggregate, platelet-rich fibrin, vital pulp therapy

## Abstract

Objectives

This study aimed to compare the clinical and radiographic outcomes of platelet-rich fibrin (PRF) and mineral trioxide aggregate (MTA) when used as direct pulp-capping agents in cariously exposed permanent teeth in adult patients.

Materials and methods

This randomized, parallel-group clinical trial included 20 permanent molars with carious pulp exposure in adults aged 18-45 years. Teeth exhibiting controlled bleeding, positive responses to pulp sensibility tests, and no radiographic evidence of periapical pathology were included. Eligible teeth were randomly allocated to receive either PRF or MTA as the direct pulp-capping material. Following placement of the capping agent, all teeth were restored using a resin-modified glass ionomer liner and resin composite restoration. Clinical evaluations, including cold test, electric pulp test, tenderness to percussion, tooth mobility, and periodontal probing depth, were performed at three and six months. Cone-beam computed tomography (CBCT) was conducted at six months to evaluate the presence and volume of dentin bridge formation.

Results

Nineteen of the 20 enrolled teeth completed the six-month follow-up (n = 19/20; 95%). CBCT analysis revealed significantly greater dentin bridge volume in the PRF group compared with the MTA group (0.01032 ± 0.00341 cc vs. 0.00778 ± 0.00120 cc; *p* = 0.049). All retained teeth in both groups demonstrated normal responses to cold and electric pulp testing (n = 19/19; 100%), with no tenderness to percussion or periodontal abnormalities. No adverse events were observed in the PRF group. One tooth in the MTA group developed symptoms of irreversible pulpitis at three months (n = 1/10; 10%) and required root canal treatment.

Conclusion

PRF demonstrated superior hard-tissue regenerative potential compared to MTA while maintaining comparable pulpal vitality. Within the limitations of this study, PRF may be considered a biologically advantageous alternative to MTA for direct pulp capping of carious exposures.

## Introduction

Vital pulp therapy (VPT) has emerged as a biologically driven approach for preserving pulpal vitality in teeth affected by deep carious lesions. Direct pulp capping (DPC), in particular, aims to maintain the structural and functional integrity of the pulp-dentin complex by promoting reparative dentinogenesis through the placement of a bioactive material directly over the exposed pulp [1,2].

Mineral trioxide aggregate (MTA) is widely regarded as a gold standard material for DPC due to its excellent biocompatibility, sealing ability, and predictable dentin bridge formation [3,4]. However, clinical limitations such as extended setting time, handling difficulties, potential tooth discoloration, and higher cost have been reported in several studies, prompting the exploration of alternative biologically active materials [5].

Platelet-rich fibrin (PRF), a second-generation autologous platelet concentrate, has gained attention due to its ability to release a sustained concentration of growth factors that promote angiogenesis, cellular proliferation, and differentiation of dental pulp stem cells. Unlike conventional materials, PRF functions as a natural scaffold and enhances the intrinsic healing potential of the pulp [6].

Carious pulp exposures in adult patients present a unique clinical challenge due to increased microbial load, deeper inflammatory changes, and comparatively reduced regenerative potential of the pulp tissue [7,8]. In such scenarios, a biologically active material capable of modulating inflammation and enhancing tissue regeneration may offer improved outcomes.

Although PRF has shown promising results in regenerative endodontics, limited clinical evidence exists comparing its effectiveness with MTA specifically in carious pulp exposures in adult permanent teeth [9,10].

Therefore, the aim of this randomized clinical trial was to compare the clinical and radiographic outcomes of PRF and MTA as DPC agents in cariously exposed permanent teeth in adults.

The primary objective of this randomized clinical trial was to compare and evaluate the extent of dentin bridge formation, in terms of its presence, continuity, and volumetric assessment using cone-beam computed tomography (CBCT), following DPC using PRF and MTA in cariously exposed permanent teeth in adult patients. The secondary objectives were to assess pulp sensibility response (cold test and electric pulp test (EPT)), clinical symptoms (pain), and the presence of any radiographic periapical changes.

Null hypothesis

There is no significant difference in the clinical and radiographic outcomes between PRF and MTA when used as DPC agents in carious pulp exposures.

## Materials and methods

Study design and ethical approval

This study was conducted as a randomized, parallel-group clinical trial comparing PRF and MTA as direct pulp capping materials in permanent teeth with carious pulp exposure. The study protocol was reviewed and approved by the Institutional Ethics Committee (IEC GDCH/CONS.2/2025) and registered in the Clinical Trials Registry-India (CTRI/2025/04/085645). Written informed consent was obtained from all participating individuals prior to enrolment.

Participants were randomly assigned to either the PRF group or the MTA group in a 1:1 allocation ratio using a computer-generated randomization sequence. The random sequence was generated by an independent investigator not involved in the clinical procedures to ensure unbiased allocation. Allocation concealment was achieved using sequentially numbered, opaque, sealed envelopes (SNOSE technique). Each envelope contained the group assignment and was opened only at the time of intervention, immediately prior to the procedure. This method ensured that both the operator and the participant remained unaware of the allocation sequence until the point of treatment, thereby minimizing selection bias. The outcome assessment was carried out by independent, calibrated evaluators who were blinded to group allocation.

To minimize the influence of potential confounding factors, strict inclusion and exclusion criteria were applied to ensure a homogeneous study population. Variables such as age, tooth type, extent of carious lesion, and pulpal status were standardized at baseline. In addition, all clinical procedures were performed by a single calibrated operator using a uniform protocol to eliminate inter-operator variability. Random allocation of participants further ensured an equal distribution of known and unknown confounders between the study groups, and outcome assessment was carried out by blinded evaluators to reduce detection bias.

Sample selection

Patients aged 18-45 years reporting to the Department of Conservative Dentistry and Endodontics were screened for eligibility. Participant recruitment and clinical procedures were conducted from April 2025, and follow-ups were conducted till December 2025. All teeth were clinically and radiographically examined prior to inclusion.

The study included maxillary or mandibular molars presenting with deep occlusal caries and exhibiting clinical signs and symptoms consistent with reversible pulpitis. Reversible pulpitis was diagnosed based on the presence of provoked pain in response to thermal stimuli, absence of spontaneous pain, positive response to electric pulp testing, and a non-lingering response to cold testing that resolved within a few seconds after removal of the stimulus. Teeth exhibiting prolonged or lingering pain (≥30 seconds) were excluded as indicative of irreversible pulpitis [11]. Teeth exhibiting spontaneous pain or showing negative responses to pulp sensibility tests were excluded. Additionally, teeth with any radiographic evidence of periapical pathology were not included in the study. Patients presenting with systemic diseases or conditions that could affect healing were not included. Teeth that were unsuitable for rubber dam isolation, previously restored teeth, or teeth exhibiting cracks or fractures were excluded. Periodontally compromised teeth and patients with bleeding disorders, abnormal bleeding time (BT), clotting time (CT), or platelet count abnormalities, and those with poor oral hygiene were also excluded. Furthermore, pregnant or lactating women were not included in the study.

Sample size calculation

A sample size estimation was performed using G*Power version 3.0.1 (Franz Faul, Universität Kiel, Germany). The Wilcoxon-Mann-Whitney test (two-tailed) was selected using the asymptotic relative efficiency (ARE) method. An effect size of d = 1.38 [8], based on the expected difference in volume of dentin bridge formation, was used as the reference value. Setting α = 0.05, power = 80%, and an allocation ratio of 1:1, a minimum of 10 teeth per group was required, resulting in a total sample size of 20. The calculated actual power was 0.811, confirming the adequacy of the study design.

Preoperative assessment

For all eligible teeth, standardized preoperative periapical radiographs were obtained. Pulp sensibility testing was standardized using an electric pulp tester (Digitest II, Parkell Inc., USA) and a cold test using refrigerant spray (Endo Ice, Coltene/Whaledent, USA). Standardized intraoral periapical radiographs were obtained using the paralleling technique with a digital sensor to ensure reproducibility and consistent angulation across all time points. Oral prophylaxis was performed prior to the operative procedure. Teeth meeting the inclusion criteria and exhibiting positive sensibility responses were scheduled for direct pulp capping.

Clinical procedure

Operative Protocol

Patients rinsed with 0.2% chlorhexidine gluconate prior to treatment. Local anesthesia was administered using 2% lignocaine hydrochloride with 1:80,000 adrenaline. All operative procedures were performed under 3x magnification using dental loupes (Zumax). Rubber dam isolation was achieved in all cases. Caries excavation was performed using sterile round carbide burs and straight fissure burs under water coolant. On approaching deeper, the remaining caries were excavated using sterile, sharp spoon excavators. Teeth that exhibited a carious pulp exposure measuring approximately 0.5-1.0 mm in diameter were considered for inclusion. The size of pulpal exposure (0.5-1.0 mm) was assessed using a calibrated periodontal probe under magnification to enhance measurement accuracy.

Hemostasis was achieved using a sterile cotton pellet moistened with 2.5% sodium hypochlorite and was considered adequate if bleeding was controlled within five minutes. This threshold is commonly used as a clinical indicator of reversible pulpal inflammation and a predictor of favorable outcomes in VPT [12,13]. The use of sodium hypochlorite not only aids in achieving hemostasis but also provides antimicrobial action and clearance of dentinal chips along with damaged cells at the exposure site, thereby creating a conducive environment for pulpal healing [12]. Teeth with uncontrolled bleeding were excluded.

PRF Group

PRF was prepared following Choukroun’s protocol [6]. Approximately 5-10 mL of venous blood was drawn from the antecubital vein under aseptic conditions and immediately transferred into sterile tubes without anticoagulant. Centrifugation was initiated immediately after blood collection to prevent premature clotting. The samples were centrifuged using a tabletop centrifuge (Model: R-303, REMI Elektrotechnik Ltd., Vasai, Maharashtra, India) at 3000 rpm for 10 minutes.

Following centrifugation, three distinct layers were obtained: an upper acellular platelet-poor plasma layer, a middle PRF clot, and a lower red blood cell layer. The PRF clot from the middle layer was carefully retrieved using sterile tweezers and gently separated from the underlying red blood cell layer using sterile scissors. The clot was then placed on a sterile glass slab and compressed using another sterile glass slab to expel excess serum, thereby forming a dense and resilient fibrin membrane.

**Figure 1:**
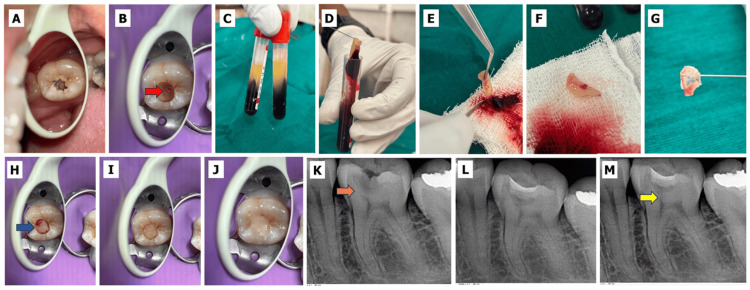
Direct pulp capping with platelet-rich fibrin. A) Preoperative clinical photograph. B) The red arrow denotes pulpal exposure evident on caries excavation. C-F) Leukocyte-rich platelet-rich fibrin (L-PRF) isolated by Choukroun’s protocol. G-H) The blue arrow denotes the PRF membrane placed over the exposure site. I) Placement of resin-modified glass ionomer cement (RMGIC). J) Final composite restoration. K) Preoperative radiograph (the orange arrow denotes deep carious lesion evident on the radiograph). L) Immediate postoperative radiograph. M) Six-month follow-up radiograph (the yellow arrow denotes the bridge formation evident on the radiograph). All clinical and radiographic images are original and were obtained during the present study.

The resulting membrane was trimmed into small pieces and transferred to the pulp exposure site using the tine of an explorer. Gentle adaptation was performed using a sterile ball burnisher to ensure intimate contact with the underlying pulp tissue. (Figure 1).

The PRF obtained using this protocol corresponds to leukocyte-rich PRF (L-PRF), characterized by a dense fibrin matrix enriched with platelets and leukocytes.

MTA Group

**Figure 2:**
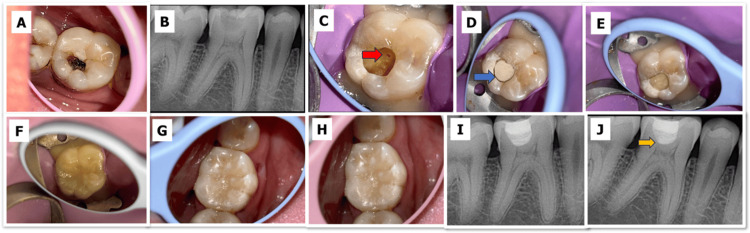
Direct pulp capping with mineral trioxide aggregate (MTA). A) Preoperative clinical photograph. B) Preoperative radiograph. C) The red arrow denotes pulpal exposure on caries excavation. D) MTA placed over the exposure site (blue arrow). E) Placement of resin-modified glass ionomer cement (RMGIC). F) Final composite restoration. G) Immediate postoperative photograph. H) Six-month follow-up clinical photograph. I) Immediate postoperative radiograph. J) Six-month follow-up radiograph (the yellow arrow denotes the bridge formation evident on the radiograph). All clinical and radiographic images are original and were obtained during the present study.

MTA (MTA Plus, Prevest DenPro Ltd.) was mixed in accordance with the manufacturer’s instructions and placed directly over the exposure site in a thickness of approximately 2 mm. The material was gently adapted, and a moist sterile cotton pellet was placed briefly to facilitate the initial setting reaction (Figure 2).

Restorative Procedure

Following placement of the pulp capping material, the cavity floor was lined with resin-modified glass ionomer cement (RMGIC) (Light-Cured Universal Restorative, GC Gold Label 2 LC, GC Corporation, Japan) in a thickness of approximately 1-2 mm and light-cured for 20 seconds according to manufacturer instructions. A universal adhesive (Scotchbond Universal Adhesive, 3M ESPE, Seefeld, Germany) was applied using a self-etch protocol to minimize additional pulpal irritation and preserve dentin integrity. The tooth was then restored using incremental light-cured resin composite (Ivoclar, Vivadent). Final restoration was adjusted for occlusion, followed by finishing and polishing with a composite finishing system (Super Snap Rainbow Technique kit, Shofu) to ensure optimal marginal integrity.

Radiographic Assessment

Standardized intraoral periapical radiographs were obtained at each recall visit to evaluate periodontal ligament (PDL) space, periapical status, and signs of hard tissue formation.

Cone-Beam Computed Tomography (CBCT) Evaluation

CBCT imaging was performed using a NewTom CBCT system (Cefla S.C., Bologna, Italy) with a limited field of view (6 × 6 cm) and a voxel size of 80 µm to minimize radiation exposure while ensuring high-resolution imaging. A single scan was obtained at the six-month follow-up in accordance with the ALARA (as low as reasonably achievable) principle. CBCT was considered essential in this study due to its superior ability to assess the continuity and volume of dentin bridge formation compared to conventional two-dimensional radiography.

The acquired DICOM datasets were analyzed using the BlueSky Bio software (version 4.0, BlueSky Bio, LLC, USA). Prior to analysis, two independent examiners underwent calibration sessions to standardize the identification and segmentation of the dentin bridge region. Inter-examiner reliability was assessed using the intraclass correlation coefficient (ICC), which demonstrated high agreement (ICC > 0.85), indicating excellent reproducibility of measurements.

**Figure 3:**
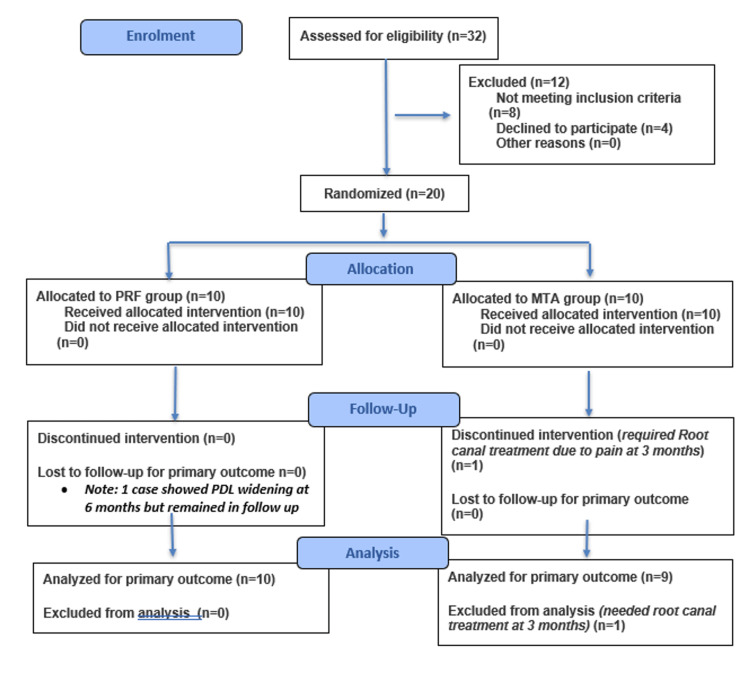
CONSORT 2025 flow diagram Flow diagram of the progress through the phases of a randomized trial of two groups (enrollment, intervention allocation, follow-up, and data analysis).

Participants were monitored for any adverse events, including postoperative pain, swelling, infection, or allergic reactions, throughout the follow-up period (Figure 3).

Postoperative Follow-Up

Of the 20 treated cases, one patient in the MTA group reported pain at the three-month recall and subsequently underwent root canal treatment. A total of 19 cases completed the six-month follow-up period. At the six-month evaluation, one tooth in the PRF group demonstrated PDL space widening on radiographic examination, although the patient remained asymptomatic with no clinical signs suggestive of irreversible pulpitis.

The primary outcome measure was the assessment of dentin bridge formation, including its presence, continuity, and quality. Secondary outcome measures included clinical parameters (pulp sensibility, tenderness, mobility, and probing depth) and radiographic findings at each recall interval. No adverse events or complications were reported in any of the treated groups throughout the follow-up period (Figure 3).

Clinical assessment

At each follow-up visit at three and six months, the treated teeth were clinically evaluated for pulp sensibility using cold testing and electric pulp testing. Periodontal probing depth, tooth mobility, the presence of pain, and tenderness to percussion were also assessed to determine pulpal status and periradicular health. All findings were recorded systematically at each recall.

Radiographic assessment

Radiographic evaluation was performed at both recall intervals using digital intraoral radiography (Sirona, Dentsply). Standardized exposure parameters of 100 ms, 4 mA, and 60 kVp were used for all images. Radiographs were examined for PDL space, periapical status, and any evidence of hard tissue formation beneath the pulp capping material, with consistent angulation and exposure maintained across visits.

CBCT assessment

The pulp-capped teeth underwent CBCT evaluation at the six-month recall using a NewTom CBCT system (Cefla S.C., Bologna, Italy). Exposure parameters were set at 90 kV and 4 mA with an approximate scan time of 18 seconds. The reconstructed DICOM datasets were exported for three-dimensional volumetric analysis of dentin bridge formation.

The use of CBCT at the six-month follow-up was justified by the need for an accurate three-dimensional assessment of dentin bridge formation, including its continuity and volume, which cannot be reliably evaluated using conventional two-dimensional radiography. To minimize radiation exposure, CBCT imaging was performed using a limited field of view (6 × 6 cm) and a single scan per patient, strictly adhering to the ALARA principle. The diagnostic benefits of obtaining precise volumetric data for the primary outcome measure were considered to outweigh the minimal radiation risk. Furthermore, all participants were informed about the procedure and provided written consent prior to imaging.

**Figure 4:**
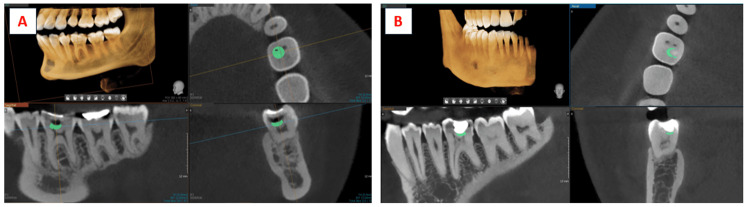
Measurement and marking of the formed dentin bridge in the A) platelet-rich fibrin (PRF) group and B) mineral trioxide aggregate (MTA) group for volumetric analysis. Dentine bridge below the exposure site marked (green) for image segmentation and 3D image generation. All radiographic images are original and were obtained during the present study.

**Figure 5:**
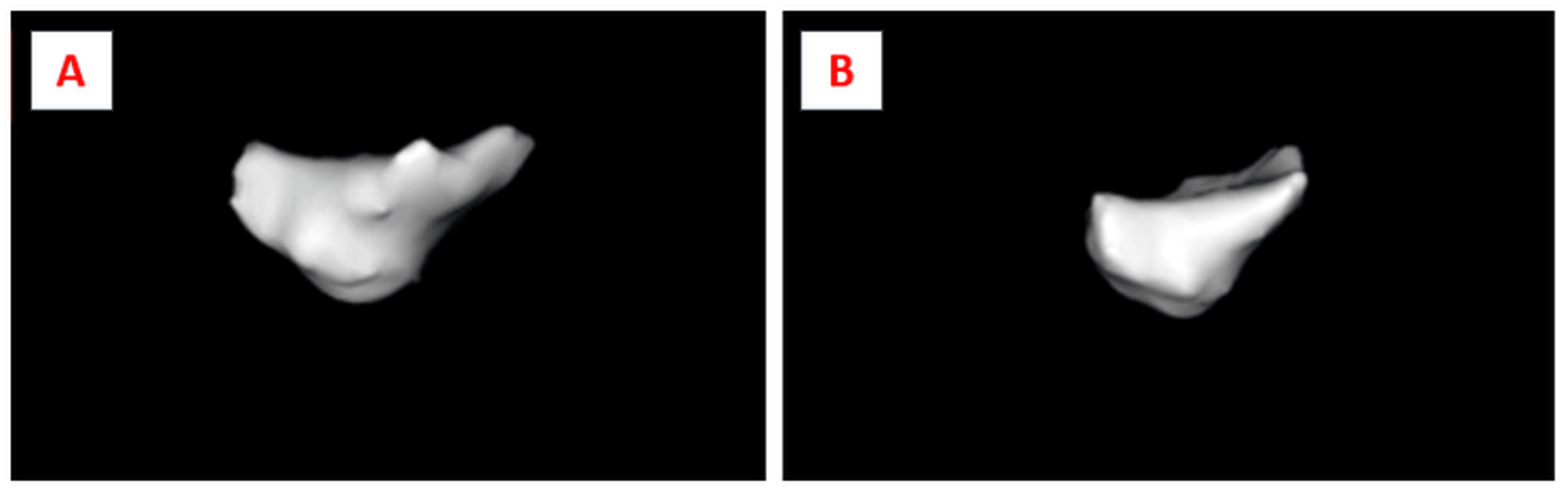
Three-dimensional reconstructed image of the dentine bridge: A) platelet-rich fibrin (PRF) group, B) mineral trioxide aggregate (MTA) group. All images are original and obtained during the present study.

The CBCT datasets were analyzed using the BlueSky Bio software (version 4.0). Interpretation of the scans was performed by two independent, calibrated assessors-one endodontist and one oral radiologist. Each case was evaluated for the presence or absence of dentin bridge formation, and the volume of the formed bridge was quantified in cubic centimeters (cc). To standardize the measurement zone, the region immediately beneath the pulp-capping site was localized by identifying the exact exposure point on serial CBCT slices. A region of interest (ROI) corresponding to the expected site of hard-tissue formation was then delineated using the software cursor (Figure 4). The selected ROI was exported in a .STP file format and subsequently visualized in the BlueSky Bio software to generate mesh data representing the formed dentin bridge for volumetric computation (Figure 5). Prior to initiating image evaluations, both assessors underwent repeated calibration sessions to ensure reliability. The same standardized protocol was used for all subjects.

Statistical analysis

Statistical analysis was performed using SPSS software version 26.0 (IBM Corp., Armonk, NY, USA). The level of statistical significance was set at p < 0.05. Descriptive statistics, including mean and standard deviation for continuous variables and proportions for categorical variables, were calculated for both groups.

The normality of continuous variables was assessed using the Shapiro-Wilk test. As the data did not follow a normal distribution and considering the small sample size, non-parametric tests were applied. Intergroup comparison of dentin bridge volume was performed using the Mann-Whitney U test.

Categorical variables, including responses to pulp sensibility tests and other clinical parameters, were compared using Fisher’s exact test. All analyses were conducted with a 95% confidence interval. Missing data were handled using complete-case analysis, and only cases that completed the six-month follow-up were included in the final statistical evaluation.

No interim analysis was performed due to the relatively short follow-up period and predefined sample size.

## Results

**Figure 6:**
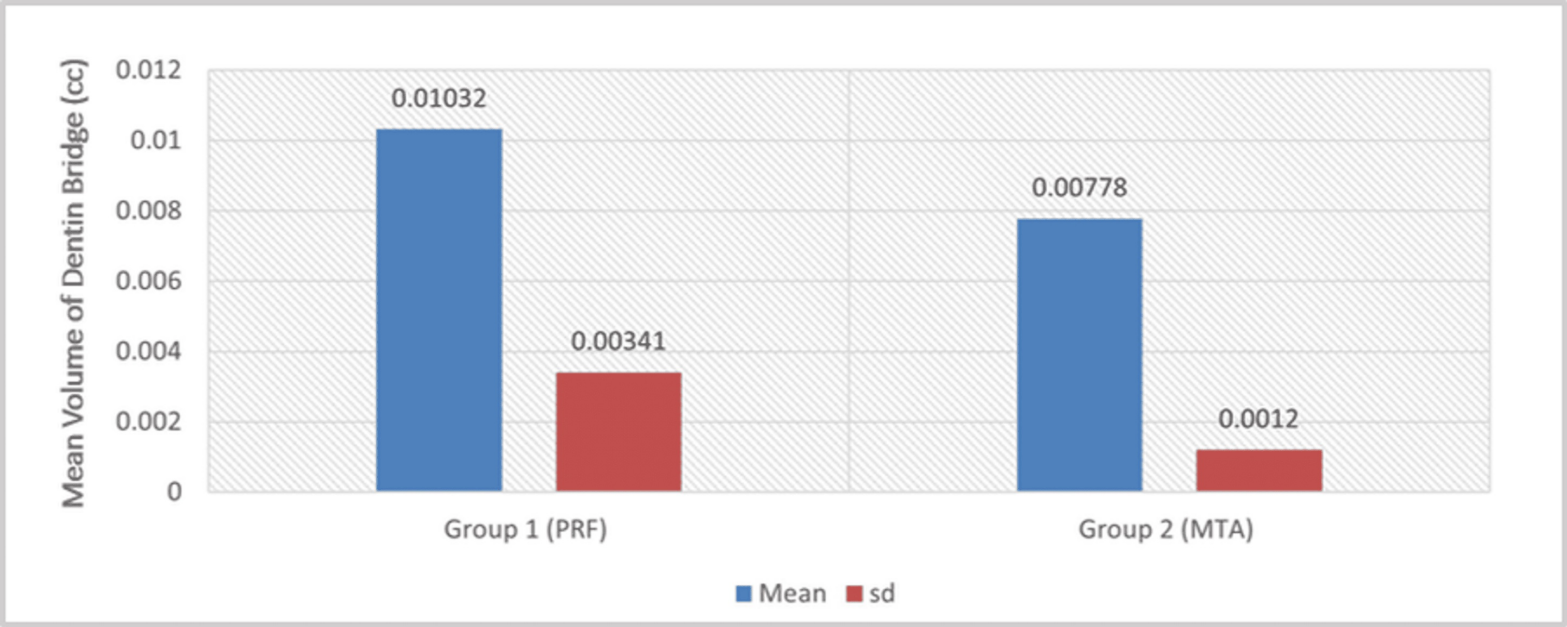
Comparison graph of the mean volume of the bridge (cc) between the PRF and MTA groups PRF: platelet-rich fibrin, MTA: mineral trioxide aggregat, sd: standard deviation

A total of 20 teeth were enrolled and randomly allocated into two groups (PRF and MTA). One patient in the MTA group reported persistent pain at the three-month review and subsequently underwent root canal therapy; therefore, 19 teeth were available for final analysis at six months (n = 19/20, 95%). All remaining teeth were clinically asymptomatic and demonstrated positive responses to pulp sensibility tests throughout the follow-up period. CBCT assessment at six months revealed that the PRF group exhibited a significantly greater volume of dentin bridge formation (0.01032 ± 0.00341 cc) compared with the MTA group (0.00778 ± 0.00120 cc), with a mean difference of 0.00254 cc that was statistically significant (p = 0.049). This indicates that PRF stimulated a more substantial early hard-tissue regenerative response beneath the exposure site (Table 1, Figure 6).

**Table 1:** Comparison of the mean difference of the dentine bridge volume (cc)

Variable	Group 1 (PRF)	Group 2 (MTA)	Mean difference	t-value	p-value
Volume of the bridge (cc)	0.01032 ± 0.00341	0.00778 ± 0.00120	0.00254	2.118	0.049*

Clinically, all teeth treated with PRF (n = 10/10; 100%) and all retained teeth in the MTA group (n = 9/9; 100%) responded normally to cold testing (Table 2) and EPT (Table 3) during both the three- and six-month evaluations (p = 0.99), confirming that PRF and MTA were equally effective in maintaining pulpal vitality over the six-month period.

**Table 2:** Comparison of the cold test results All teeth responded positively to the cold test (n = 19/19; 100%). Chi-square test = 0.0; p-value = 0.99. PRF: platelet-rich fibrin, MTA: mineral trioxide aggregate

Cold test	PRF (n)	MTA (n)	Total
Positive	10 (100%)	9 (100%)	19 (100%)

**Table 3:** Comparison of the electric pulp test results All teeth responded positively to the electric pulp test (n = 19/19; 100%). Chi-square test = 0.0; p-value = 0.99.

Electric pulp test	PRF (n)	MTA (n)	Total
Normal	10 (100%)	9 (100%)	19 (100%)

## Discussion

The present randomized clinical trial compared the clinical and radiographic outcomes of PRF and MTA as direct pulp-capping agents in permanent teeth with carious pulp exposures in adults. Our findings demonstrated that PRF produced significantly greater dentine bridge volume compared to MTA in the direct pulp capping of cariously exposed permanent teeth. This aligns with evidence from recent systematic reviews, which have consistently shown that platelet concentrates, particularly PRF, enhance dentinogenic potential and pulpal healing through their biologically active composition [9,10].

The superior performance of PRF observed in the present study may be attributed to its three-dimensional fibrin matrix enriched with platelets and leukocytes, which serves as a biologically active scaffold for tissue repair [13]. PRF is known to release a range of growth factors, including transforming growth factor-β (TGF-β), platelet-derived growth factor (PDGF), and vascular endothelial growth factor (VEGF), in a sustained manner over time [7,8]. This gradual release supports key regenerative processes, such as angiogenesis, cellular proliferation, and differentiation of odontoblast-like cells, which are essential for reparative dentinogenesis. In addition to providing structural support, the fibrin network facilitates cell migration and extracellular matrix deposition, thereby promoting the formation of a more organized and continuous dentin bridge [14,15]. The presence of leukocytes within PRF also contributes to the modulation of the inflammatory response, creating a favorable environment for pulpal healing.

More specifically, in the present study, the platelet concentrate utilized corresponds to leukocyte-rich PRF (L-PRF), which differs from other platelet derivatives in terms of its structural and biological characteristics. L-PRF exhibits a dense fibrin architecture that enables a sustained and prolonged release of growth factors, thereby enhancing its regenerative potential. The presence of leukocytes further contributes to immunomodulation and antimicrobial activity, creating a favorable microenvironment for pulpal healing. These characteristics may explain the enhanced dentin bridge formation observed in the L-PRF group. Similar findings have been reported in previous clinical investigations, where the use of PRF in vital pulp therapy resulted in significantly greater dentin bridge formation compared to MTA, despite comparable overall clinical success rates [6].

By contrast, MTA facilitates dentin bridge formation primarily through the release of calcium ions, which interact with tissue fluids to form a hydroxyapatite layer at the material-dentin interface. This bioactive surface promotes cell attachment and contributes to its well-documented sealing ability and biocompatibility [16,17]. MTA has been shown to support odontoblast-like cell differentiation and hard tissue barrier formation, making it a reliable material for vital pulp therapy [16]. However, its mechanism of action is largely dependent on physicochemical processes rather than sustained biological signaling. As a result, dentin bridge formation with MTA, although predictable, may occur more gradually during the early healing phase [18,19], as also observed in the present study, where the volume of dentin bridge formation was comparatively lower than that seen with L-PRF.

Clinically, while MTA demonstrated successful maintenance of pulpal vitality in the majority of cases, its practical limitations, such as extended setting time, handling difficulty, and potential discoloration, may influence its routine use. These factors, although not directly affecting biological outcomes, remain relevant considerations in clinical decision-making [20,21].

Importantly, the present study observed a single treatment failure in the MTA group, where one tooth developed symptoms of irreversible pulpitis at the 3-month follow-up (n = 1/10; 10%). While MTA is widely regarded as a reliable material for vital pulp therapy, isolated failures may occur in clinical practice. In the present study, no definitive cause for this outcome could be established based on the available clinical and radiographic findings.

It is recognized that carious pulp exposures are associated with varying degrees of pulpal inflammation and bacterial involvement, which may influence healing outcomes [22,23]. However, given the limited sample size and the occurrence of only a single failure, it is not possible to attribute this outcome to any specific biological or procedural factor. Therefore, this finding should be interpreted with caution and does not necessarily reflect a limitation of the material itself.

In one L-PRF-treated case (n = 1/10; 10%), mild widening of the PDL space was observed at the six-month evaluation; however, the tooth remained completely asymptomatic and exhibited normal responses to sensibility testing. Such isolated radiographic findings are well documented in the vital pulp therapy literature and are often considered part of the normal healing spectrum rather than indicators of failure [21]. Cariously inflamed pulps may demonstrate transient periodontal or periapical remodeling during early healing, even when pulpal vitality is maintained [24,25]. Furthermore, radiographic PDL changes have been shown to persist despite histologic evidence of pulpal recovery, highlighting that PDL widening alone lacks diagnostic specificity, particularly during the early healing phase. L-PRF’s biologically active matrix may also enhance local vascular and cellular activity, contributing to subtle radiographic variations that do not reflect pathologic deterioration [26]. Therefore, the observed PDL widening in this case is best interpreted as a transient reactive change rather than a sign of adverse outcome.

The increased dentin bridge volume observed in the L-PRF group in the present study is consistent with findings reported in previous clinical studies evaluating platelet concentrates in vital pulp therapy [9,10,27,28]. This suggests that L-PRF may enhance early hard tissue formation following direct pulp capping. The underlying biological basis for this effect has been attributed to the ability of L-PRF to promote odontoblastic differentiation and mineralization, as demonstrated in experimental studies showing increased expression of dentinogenic markers such as dentin sialophosphoprotein (DSPP) and alkaline phosphatase [8,10]. These findings support the concept that L-PRF may facilitate a more organized reparative dentinogenesis rather than the formation of a less structured calcific barrier. In addition, the autologous nature of L-PRF minimizes the risk of adverse tissue reactions and avoids the potential cytotoxic effects associated with certain materials during their setting phase, further contributing to a favorable healing response [10,28,29].

CBCT analysis in the present study provided three-dimensional insight into the extent and volume of dentin bridge formation, allowing a more detailed evaluation compared to conventional two-dimensional radiography. This facilitated objective assessment of the continuity and thickness of the mineralized barrier [30]. However, the use of CBCT must be interpreted with consideration of its inherent limitations, including higher radiation exposure compared to intraoral radiography, increased cost, and ethical concerns regarding its routine use. In the present study, these concerns were addressed by limiting imaging to a single scan with a small field of view, in accordance with the ALARA principle. Despite its advantages in research settings, CBCT may not be routinely indicated for clinical evaluation of direct pulp capping outcomes, and its use should be justified based on the diagnostic need [31]. Therefore, while CBCT enhanced the accuracy of volumetric assessment in this study, its application should be interpreted within the context of these limitations.

Clinical implications

The findings of the present study suggest that L-PRF may serve as a promising biologically driven adjunct in direct pulp capping procedures. Its ability to enhance dentin bridge formation while maintaining pulp vitality highlights its potential role in regenerative vital pulp therapy. The autologous nature of L-PRF, along with its excellent biocompatibility and sustained release of growth factors, makes it particularly advantageous in clinical situations where enhanced biological healing is desired. Furthermore, its application may be beneficial in cases with higher regenerative potential, where preservation of pulp vitality is critical. While MTA remains a reliable material due to its well-established sealing ability and predictable clinical outcomes, the adjunctive use of L-PRF may further enhance early healing responses and dentinogenesis. Therefore, L-PRF may be considered as a potential adjunct or alternative in minimally invasive pulp therapy approaches.

While the results are promising, it must be acknowledged that the sample size was modest, although determined based on power calculation for the primary outcome measure. The study was adequately powered to detect differences in dentin bridge volume; however, it may not have been sufficiently powered to identify differences in secondary clinical outcomes such as pulp sensibility responses and other clinical parameters. In addition, the relatively small sample size increases the risk of type II error, where potential differences between groups may not have reached statistical significance. The six-month follow-up period provides meaningful insight into early dentinogenesis; however, it does not allow assessment of long-term pulpal survival and durability of the dentin bridge. Therefore, further studies with larger sample sizes and extended follow-up periods are recommended to validate these findings and to better evaluate long-term clinical outcomes, particularly when comparing biologically active materials, such as PRF with conventional bioactive cements like MTA.

## Conclusions

Within the limitations of this randomized clinical trial, PRF demonstrated superior dentine bridge formation and improved clinical outcomes relative to MTA in the direct pulp capping of cariously exposed permanent teeth. PRF’s sustained release of bioactive growth factors and its autologous fibrin scaffold appear to favor a more robust regenerative response and preservation of pulpal vitality. These findings support the use of PRF as a biologically advantageous alternative to traditional bioceramic materials in minimally invasive vital pulp therapy. Further studies with larger sample sizes and long-term follow-up are recommended to validate these outcomes and assess the longevity of PRF-mediated pulpal healing.
